# Fuzzy-Logic Based Distributed Energy-Efficient Clustering Algorithm for Wireless Sensor Networks

**DOI:** 10.3390/s17071554

**Published:** 2017-07-03

**Authors:** Ying Zhang, Jun Wang, Dezhi Han, Huafeng Wu, Rundong Zhou

**Affiliations:** 1 College of Information Engineering, Shanghai Maritime University, Shanghai 201306, China; yingzhang@shmtu.edu.cn (Y.Z.); dzhan@shmtu.edu.cn (D.H.); rd_shmtu@163.com (R.Z.); 2 Department of Electrical and Computer Engineering, University of Central Florida, Orlando, FL 32816, USA; 3 Merchant Marine College, Shanghai Maritime University, Shanghai 201306, China; hfwu@shmtu.edu.cn

**Keywords:** non-uniform distribution, distributed clustering, load balance, TSK fuzzy model, neighbor nodes’ energy

## Abstract

Due to the high-energy efficiency and scalability, the clustering routing algorithm has been widely used in wireless sensor networks (WSNs). In order to gather information more efficiently, each sensor node transmits data to its Cluster Head (CH) to which it belongs, by multi-hop communication. However, the multi-hop communication in the cluster brings the problem of excessive energy consumption of the relay nodes which are closer to the CH. These nodes’ energy will be consumed more quickly than the farther nodes, which brings the negative influence on load balance for the whole networks. Therefore, we propose an energy-efficient distributed clustering algorithm based on fuzzy approach with non-uniform distribution (EEDCF). During CHs’ election, we take nodes’ energies, nodes’ degree and neighbor nodes’ residual energies into consideration as the input parameters. In addition, we take advantage of Takagi, Sugeno and Kang (TSK) fuzzy model instead of traditional method as our inference system to guarantee the quantitative analysis more reasonable. In our scheme, each sensor node calculates the probability of being as CH with the help of fuzzy inference system in a distributed way. The experimental results indicate EEDCF algorithm is better than some current representative methods in aspects of data transmission, energy consumption and lifetime of networks.

## 1. Introduction

Recently, with the development of wireless communication and the low power RF (Radio Frequency) designs widely used in sensor nodes, wireless sensor networks (WSNs) have received great attention due to their wide usage in environmental monitoring, transportation, disaster rescue and homeland security [1,2]. WSNs are composed of many sensor nodes with both data collection and data forwarding capabilities. As those nodes in the network are large scale, with limited battery power and deployed randomly, a consensus has been formed that clustering routing algorithm is an energy-efficient method to handle the energy consumption and topology control problems for this kind of network. In this scheme, each cluster contains cluster head (CH) node and member nodes created by some kinds of election mechanism. Member nodes in the cluster forward information to CH node by multi-hop communication, and the relay nodes are selected by taking advantage of AODV (Ad hoc On-demand Distance Vector Routing) mechanism for routing discovery [3,4]. Compared with direct communication to CHs from member nodes, multi-hop communication within cluster can reduce the number of communication links and avoid the communication congestion because the CH communicates with more member nodes simultaneously. Moreover, multi-hop communication can let member nodes help CH to share the work of data fusion and efficiently decrease energy consumption of CHs, which will be helpful for the lifetime extension of the networks. CHs are responsible for gathering the collected information from all the member nodes. After data fusion, the data in CHs will be forwarded to the base station (BS) by single hop to achieve the data upload [5].

In clustered network system, nodes are usually deployed as non-uniform distribution with different energy consumptions and different distances between each other. If we divide them into the same scale clusters, it will always lead to uneven energy consumption, especially for some CH nodes. Thus, for the sake of load balancing in the system, we usually choose unequal clustering algorithm for WSNs. Unlike the centralized optimization, the distributed clustering algorithm does not depend on the global topology of the networks, and the node can implement the information analysis only depending on the relative information of itself and its neighbor nodes, which greatly reduces the unnecessary overhead of communication with the base station compared with centralized algorithms. Thus, this type of scheme is more reasonable to be used in WSNs currently.

In clustering routing algorithm, there is no doubt that CH nodes should consume much more energy than the member nodes. However, during the intra-cluster multi-hop communication, the nodes that are close to CH have to undertake more duties of data receiving and forwarding to the CH than the farther nodes, which may lead to more extra energy consumption. As a result, this extra consumption would reduce the lifetime of the corresponding relay nodes and have a serious influence on load balancing of the whole sensor network [6]. Especially for monitoring networks such as underwater wireless sensor networks, if the relay nodes within the cluster die prematurely due to insufficient residual energy during each round, the further associated nodes have to do routing discovery once more and reestablish the routing link, which will cause more extra energy consumption. It will destroy the stability of communication and weaken the performance of network systems. Thus, it can be seen that the residual energy of the neighbor nodes of the CHs also plays an important role in the data transmission process actually.

When designing the distributed clustering algorithm for WSNs, many factors such as node energy, node degree, and the energy situation for the surrounding neighbor nodes may all need to be considered momentarily. Therefore, how to elect the appropriate CH under the multi-condition equilibrium makes a big influence on the stability of the whole clustered networks. However, a fuzzy logic system can just provide an appropriate solution for this kind of multi-factor evaluation problem like CHs election [7]. In other words, the fuzzy logic system can integrate various clustering factors for CHs election.

**Figure 1:**
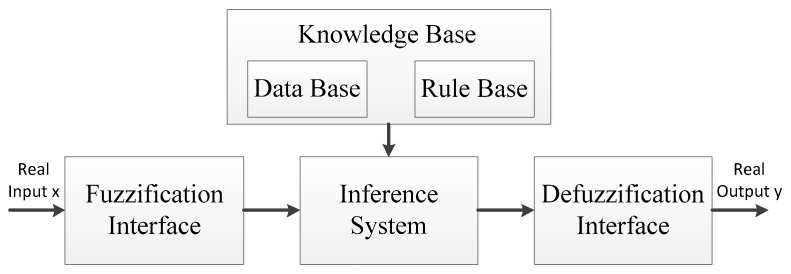
Fuzzy logic system which is composed of fuzzification interface, inference system, knowledge base and defuzzification interface.

To overcome some weakness such as the lack of accuracy when modeling some complex and high-dimensional systems, we now widely use scatter fuzzy partitions instead of the classical grid-based ones to do fuzzy analysis, in such a way that every single rule has its own meaning [8,9]. The framework of the fuzzy logic system is shown in Figure 1. We convert crisp parameters such as the current residual energy in sensor nodes into the input of fuzzy linguistic variables through fuzzification interface, and the process in defuzzification interface is carried out on the contrary. The middle part in this system is mainly composed of inference system and knowledge base. The former is used to implement the function converting the input of fuzzy linguistic variables into the system output of crisp values. The latter has two components: the database, which contains the membership function of the fuzzy partitions associated to the input linguistic variables, and the rule base, which contains the necessary if-then rules in the fuzzy analysis.

Most fuzzy systems use inference method proposed by Mamdani [10] in which the both premise and consequent parameters are defined by fuzzy sets. Mamdani-method lays the theoretical foundation of fuzzy logic and its fuzzy rules have the form shown as follows:

$$\begin{array}{l} If\;\;x_{1}\;\;is\;A_{1j}(m_{1j},\sigma_{1j})\;and\;\;\;x_{2}\;is\;A_{2j}(m_{2j},\sigma_{2j})\;\cdots \;and\;\;\;x_{n}\;is\;A_{nj}(m_{nj},\sigma_{nj})\\ Then\;\;y\;is\;B_{j}(m_{j},\sigma_{j}) \end{array}$$

where $m_{nj}$ and $\sigma_{nj}$ represent a Gaussian membership function with mean and deviation, respectively, with the *n*th dimension and the *j*th rule [11]. It translates the consequence parameter of the if-then rules into a fuzzy set as output variable. In addition, Takagi, Sugeno and Kang introduced a modified inference scheme named TSK fuzzy model [12], and its first two steps, fuzzifying the inputs and performing the fuzzy inference, are the same as the original Mamdani model. However, this kind of TSK fuzzy model defines a linear combination of the crisp inputs instead of fuzzy sets to be used as the consequent parameters. The standard of fuzzy rules of TSK fuzzy model is shown as follows:

$$\begin{array}{l} If\;\;x_{1}\;\;is\;A_{1j}(m_{1j},\sigma_{1j})\;and\;\;x_{2}\;is\;A_{2j}(m_{2j},\sigma_{2j})\;\cdots \;and\;\;\;x_{n}\;is\;A_{nj}(m_{nj},\sigma_{nj})\\ Then\;\;\;y=p_{0j}+p_{1j}x_{1}+\;\cdots \;+p_{nj}x_{n} \end{array}$$

where $p_{nj}$ represents the influence degree of each input parameter in the system. Since the consequent of a rule is crisp, the defuzzification step becomes obsolete in the TSK inference scheme. Instead, the model output is computed as the weighted average of the crisp rule outputs. This computation is more succinct than the defuzzification of the original centroid method. TSK fuzzy model is more conducive to the quantitative study for the whole system compared with Mamdani method.

Therefore, this paper proposes a distributed clustering algorithm EEDCF based on TSK fuzzy model for WSNs to cope with the problems mentioned above. For each node, we consider node’s residual energy, node’s degree and residual energy of node’s neighbor nodes as the input parameters to calculate the probability of being CH by TSK fuzzy model in a distributed way when CH election occurs.

## 2. Related Works

LEACH (Low Energy Adaptive Clustering Hierarchy) is one of the most classical self-organizing adaptive clustering routing algorithms for WSNs in the early time, which averages the energy load of the whole network to each sensor node through regular CH election [13]. It can help to improve the scalability and robustness of dynamic networks to some extent. In the CH election stage, each node will generate a random number between 0 and 1. If this random number is less than the threshold *T*(*n*), the node will be elected as the CH node. The definition of *T*(*n*) is influenced by the parameters such as probability to be the CH, current round and the percentage of allowed CHs in the whole networks, which is shown as Equation (1).

(1) $$T(n)=\left\{ \begin{array}{ccc} \frac{p}{1-p[r\mathrm{mod}(1/p)]} & , & n\in G\\ 0 & , & other \end{array}\right.$$

where *p* indicates the percentage of nodes to be CH during the election, *r* is the current CH election round and *G* is the set of nodes which fail to be selected as CH in the recent 1/*p* rounds. Here the probability of each node to be CH is the same. After that, CHs allocate time slot to the member nodes in the cluster, and the data transmission within cluster will be performed with TDMA (Time Division Multiple Access) manner.

Although LEACH is too ideal to fully consider the communication consumption in the actual transmission environment, and the randomness of CH election causes many deficiencies, LEACH provides a good theoretical basis and design model for the subsequent clustering algorithms.

HEED (Hybrid and Energy-Efficient Distributed clustering approach) sets up a primary clustering parameter and a secondary clustering parameter during CH election as a measure of the cost of intra-cluster communication [14]. The primary parameter is dependent on the residual energy of the nodes and the nodes with higher residual energy will have a higher probability of being a CH. The secondary parameter reflects nodes proximity or the nodes density, which is used as an auxiliary indicator for the computation of communication cost within the cluster. This scheme builds clusters in a distributed way, extends the lifetime of the networks through minimizing the intra-cluster communication energy consumption, and keeps a good distribution for the CH nodes. Nevertheless, there are much more messages needed to be broadcast in the stage of cluster formation, which results in more additional system energy consumption. EADEEG (Energy-Aware Data Energy-Efficient Gathering protocol for wireless sensor networks) proposes an energy-efficient data gathering protocol based on cluster structure, which is also a kind of distributed clustering algorithm [15]. This scheme considers the node’s energy and its neighbor nodes’ energies simultaneously in CH election. It can reduce the energy consumption and prolong the lifetime of the networks to some extent. However, the scheme ignores the aspect of nodes degree, which might easily lead to isolated point problems in the networks. DSBCA (Distributed Self-organization in load-Balanced Clustering Algorithm for wireless sensor networks) defines the cluster radius threshold to achieve unequal clustering [16]. In CH election phase, new CH nodes are elected based on figuring out the weights determined by residual energy and node connectivity of each member node, which are evaluated by the former CHs. Although it could obtain the optimal CHs, the energy consumption of the network will be increased aggravatingly. EEREG (Energy Efficient Routing protocol based on Evolutionary Game theory) figures out the reasonable size of each cluster and the suitable CHs through game theory [17]. However, it is a kind of centralized algorithm with the awareness of the global topology of the networks, and also it will need higher requirements for the hardware of the sensor nodes.

CHEF (Cluster Head Election mechanism using Fuzzy logic in wireless sensor networks) is a kind of clustering algorithm which introduces fuzzy logic into wireless sensor networks to optimize the energy consumption of the system [18]. CHEF chooses nodes residual energy and distance between nodes and base station as the input values to compute the probability of each node to be CH. Although the algorithm offers a good decision scheme for wireless sensor networks in distributed CH election, it ignores the influence of nodes degree on the energy utilization efficiency for clustered networks. DFLC (Distributed Fuzzy Logic-based Clustering algorithm) is also a distributed clustering algorithm using fuzzy approach for WSNs [19]. It decreases the number of unnecessary data packages which the relay nodes need to receive and forward during the messages transferring from the leaf nodes to the root node. Moreover, it eliminates the message of the nodes which have a lower probability to be a new root. In other words, it adds a filter mechanism before CHs election to improve the quality of the candidate nodes. In this algorithm, it chooses node residual energy, distance to the base station and nodes density as consideration factors for the input of fuzzy approach. The output parameter “probability” is a fuzzy linguistic variable with five stages of fuzzy division. The output is converted to crisp value by Center of Area (COA) method. However, it ignores the hotspots problem in multi-hop communication, and it may cause load imbalance and lead to an earlier death of relay nodes, which affects the overall performance of the whole network systems. Moreover, considering the distance between nodes and BS is not quite suitable in dynamic network because the location of nodes is uncertain in dynamic networks, it will cause a lot of energy consumption if all the nodes have to communicate with the BS to get the current distances in real time in each round. LAUCF (Low-energy Adaptive Unequal Clustering protocol using Fuzzy c-means) is a kind of low energy adaptive unequal clustering algorithm using fuzzy c-means to select suitable CH nodes to uniform energy consumption [20]. In [21], the authors also proposed a fuzzy c-means clustering algorithm to save energy consumption. Cluster head is selected based on sensor’s location within each cluster, its location with respect to fusion center (FC), its signal-to-noise ratio (SNR) and its residual energy. In [22], the authors made use of fuzzy c-means to cluster network to reduce the shortest path error between far nodes and improve the final localization accuracy for irregular cognitive radio networks localization. Although making use of c-means can get more reasonable fuzzy analysis results, nodes require much more extra energy consumption during every CH election in this way. DUCF (Distributed Unequal Clustering using Fuzzy logic) is a distributed clustering algorithm based on fuzzy logic for unequal clustering networks, which chooses node residual energy, node degree, and the distance between nodes and base station as the input, and chooses the probability to be elected as the CH and the size of the cluster as the outputs [23]. This scheme not only takes the node’s own factors, but also the cluster size into consideration, and enhances the overall performance of the networks. Similar to the shortages in DFLC, getting the distance between nodes and BS in real time also leads to a lot of energy consumption in dynamic network, which has a negative influence on the lifetime of sensor nodes.

Considering the inadequacy of the algorithms mentioned above, we propose an Energy Efficient Distributed Clustering algorithm using Fuzzy approach for wireless sensor networks with non-uniform distribution called EEDCF. The algorithm is a fully distributed clustering algorithm that each node uses TSK fuzzy inference system to analyze whether it fits to be the CH compared with its neighbor nodes. Nodes only need to communicate with the neighbor nodes to maintain the local topology. We define the input parameters as node residual energy, node degree and neighbor nodes’ residual energies. Fuzzy logic is appropriate for making real-time decisions with multi-causal factors in the absence of complete environment information for sensor nodes in the network. We introduce neighbor nodes’ residual energies as the additional input value which can optimize the load balance of relay nodes, avoid the hotspots problem caused by multi-hop communication and it will be more helpful to the optimization of energy consumption and extend the lifetime of the network system.

## 3. System Model

### 3.1. Network Model and Hypothesis

In our scheme, there are many nodes with unique ID deployed randomly in the designated network area. Nodes are heterogeneous in terms of energy and the death of the node is only related to the energy depletion [2,24]. The Base Station (BS) is fixed after deployment. The location of the base station is aware to all nodes and the energy of the base station is assumed unlimited. The distance between nodes is calculated by using received signal strength indicator (RSSI) and data transmission between member nodes and CHs is with multi hop communication. The nodes only know the relevant information of neighbor nodes and do not know the exact situation of global topology. The communication environment of MAC layer is ideal.

### 3.2. Energy Model

Different from the wired network routing algorithms which focus on transmission bandwidth, WSN is an energy heterogeneous network and the routing algorithm pays more attention to the energy consumption of the whole system [25]. Therefore, the actual communication channel loss model plays an important role in the design of routing algorithm. The wireless transmission energy model used in this paper is referred to [26]. When Nodes transmit *k* bit data to a location to where the distance is *d*, the energy consumption composed of the transmission circuit loss and power amplification is as shown in Equation (2).

(2) $$E_{TX}\left(k,d\right)=\left\{ \begin{array}{ll} k\cdot E_{elec}+k\cdot \xi_{fs}\cdot d^{2}, & d<d_{0}\\ k\cdot E_{elec}+k\cdot \xi_{mp}\cdot d^{4}, & d\geq d_{0} \end{array}\right.$$

where $E_{elec}$ represents the energy dissipation to run the radio electronics, $\xi_{fs}$ and $\xi_{mp}$ are the energy coefficients needed to run the amplifier for two kinds of distances (close and far).

$d_{0}$ is the distance threshold value shown in Equation (3).

(3) $$d_{0}=\sqrt{\xi_{fs}/\xi_{mp}}$$

The energy consumption required by the node to receive *k* bits data is shown in Equation (4).

(4) $$E_{RX}(k)=k\cdot E_{elec}$$

After receiving information from member nodes, the energy consumption that the CH has to spend to data fusion is $E_{DA}$ when handling with 1 bit data.

## 4. EEDCF Algorithm

Fuzzy logic can be applied to handle the evaluation problems with complicated factors and conditions via simulating human beings’ making decision. It provides a feasible method to achieve the conclusion according to the descriptive language to deal with the input data like human operators, which is suitable to be used for CH election [27].

The proposed EEDCF algorithm combines fuzzy logic with clustering algorithm for wireless sensor networks and considers node residual energy, node degree, and neighbor nodes’ energy as input values for each node, which are defined as follows:

Node residual energy: The current energy of each node. Due to the need to undertake data gathering mission within the cluster and communicate with the BS, the energy consumption of CH is far more than the other member nodes in the cluster. Therefore, selecting the nodes with higher residual energy to be the CHs can greatly improve the performance of the whole system and increase the lifetime of the networks.

Node degree: The number of neighbor nodes within communication radius “R”. The greater the node degree is, the higher the efficiency of data transmission will be, and the smaller the shared workload of data forwarding taken on by each neighbor node of CH will be also, which is good for the entire system energy optimization.

Neighbor nodes’ average residual energy: Due to the multi-hop communication model for data transmission within the cluster, the nodes closer to the CH need more energy to implement data forwarding than the farther ones. Thus, there is no doubt that introducing neighbor nodes’ average residual energy as one of the decision factors is an effective solution to the load balancing problem for the whole network system.

In this paper, we define four states for the nodes: (1) initial state; (2) competing CH state; (3) elected CH state; and (4) member node state. Table 1 shows the message format and description used in the EEDCF algorithm, and the pseudo code of the proposed algorithm describing every CH election process is shown in Algorithm 1.

The cluster formation process can be divided into three phases: (1) information table updating; (2) CH election; and (3) cluster build. Those phases are described in the upcoming subsections.

**Algorithm 1.** The Proposed Clustering Algorithm for EEDCF1 **Begin** 2 *N=Total number of nodes* 3 *i=ID of living sensor node in current round* 4 *node[i].statement=initial_state* 5 **for each**
*node[i]* 6  *receives Node_MSG from Neighbor_Node* 7  *node[i].Info_table updates* *▷Input parameters: Residual Energy (RE), Node Degree (ND), Neighbor nodes’ Residual Energy (NRE)* 8  *node[i].RE=residual energy of node[i]* 9  *node[i].ND=number of nodes within communication “R*” 10  *node[i].NRE=residual energy of neighbor nodes of node[i]* *▷Analysis through fuzzy inference system (FIS)* 11  *probability=FIS(node[i].RE, node[i].ND, node[i].NRE)* 12  *Send Head_compete to all neighbor nodes* 13  *Neighbor_Node[j]=list of Head _compete from neighbor node* *▷Comparing the result from FIS (Fuzzy Inference System) with neighbor nodes’* 14   **If**
*(node[i].probability> Neighbor_Node[j].probability)* 15      *node[i].statement=CH*
16      *advertise CH_Message* 17    **else** 18       *on receiving CH_Message* 19       *select the nearest CH* 20       *send Node_JOIN to the nearest CH* 21    **end** 22 **end** 23 **End**

### 4.1. Information Table Updates Phase

Each sensor node stores an information table that contains the current node ID, the current node energy, neighbor nodes’ ID, and their corresponding residual energies. After nodes’ communication within radius “R” by sending Node_MSG message, each sensor node obtains neighbor nodes’ relevant information and updates its Info_table immediately. Then for each node, we can get the node degree *d* and calculate the neighbor nodes’ average residual energy $E_{a}$ as shown in Equation (5).

(5) $$E_{a}=\frac{\sum Neighbor\_Node_{id}.E_{residual}}{d}$$

After that, we can get the moment *t* when the CH election message namely Head_compete is sent, which can be calculated as Equation (6) [28].

(6) $$t=k\cdot T\cdot \frac{E_{a}}{E_{residual}}$$

where *k* is a random number between 0.9 and 1, *T* is the pre-specified initial duration set up in the CH election algorithm, and $E_{residual}$ indicates the node’s current residual energy.

### 4.2. Cluster Head Competition Phase

After updating the information table, each node chooses node residual energy (NE), node degree (ND), and neighbor nodes’ average residual energy (NNE) as the input parameters and converts them into fuzzy linguistic variables to perform the fuzzy logic analysis, and figure out the performance evaluation of the node. Their fuzzy sets are shown as Equation (7).

(7) $$\begin{array}{l} A_{1}(NE)=\left\{X_{1}=“low”,X_{2}=“medium”,X_{3}=“high”\right\}\\ A_{2}(ND)=\left\{X_{1}=“less”,X_{2}=“average”,X_{3}=“enormous”\right\}\\ A_{3}(NNE)=\left\{X_{1}=“weak”,X_{2}=“normal”,X_{3}=“strong”\right\} \end{array}$$

As we can see, the fuzzy linguistic variable for node residual energy has the membership degree division as: “*low*”, “*medium*” and “*high*”, the fuzzy linguistic variable for node degree has the membership degree division as: “*less*”, “*average*” and “*enormous*”, and the fuzzy linguistic variable for neighbor nodes’ average residual energy has the membership degree division as: “*weak*”, “*normal*” and “*strong*”.

(8) $$\mu_{TRI}(x)=\left\{ \begin{array}{ll} 0 & x\leq a_{1}\\ \frac{x-a_{1}}{b_{1}-a_{1}} & a_{1}\leq x\leq b_{1}\\ \frac{c_{1}-x}{c_{1}-b_{1}} & b_{1}\leq x\leq c_{1}\\ 0 & c_{1}\leq x \end{array}\right.$$

(9) $$\mu_{TRA}(x)=\left\{ \begin{array}{ll} 0 & x\leq a_{2}\\ \frac{x-a_{2}}{b_{2}-a_{2}} & a_{2}\leq x\leq b_{2}\\ 1 & b_{2}\leq x\leq c_{2}\\ \frac{d_{2}-x}{d_{2}-c_{2}} & c_{2}\leq x\leq d_{2}\\ 0 & d_{2}\leq x \end{array}\right.$$

**Figure 2:**
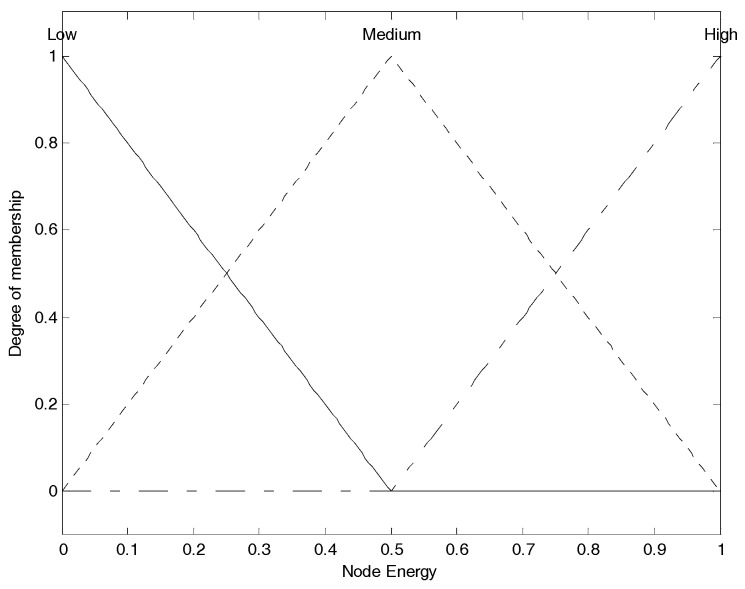
Nodes energy membership function.

**Figure 3:**
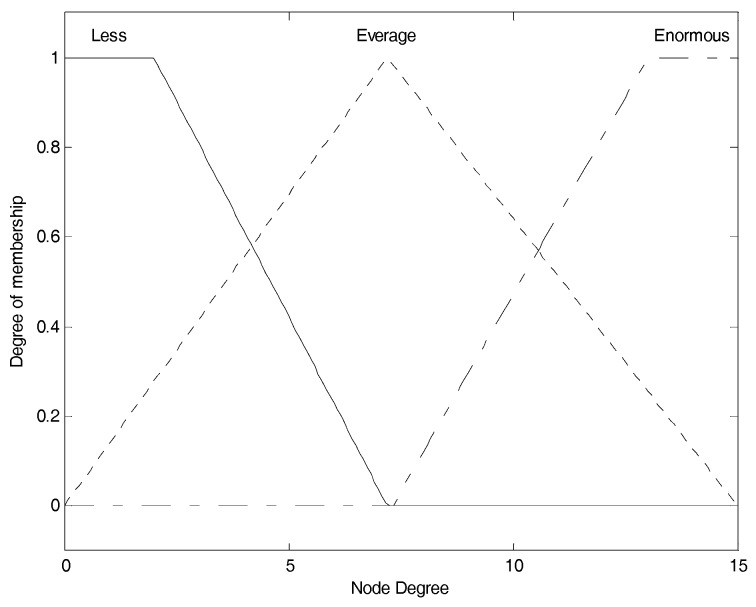
Nodes degree membership function.

**Figure 4:**
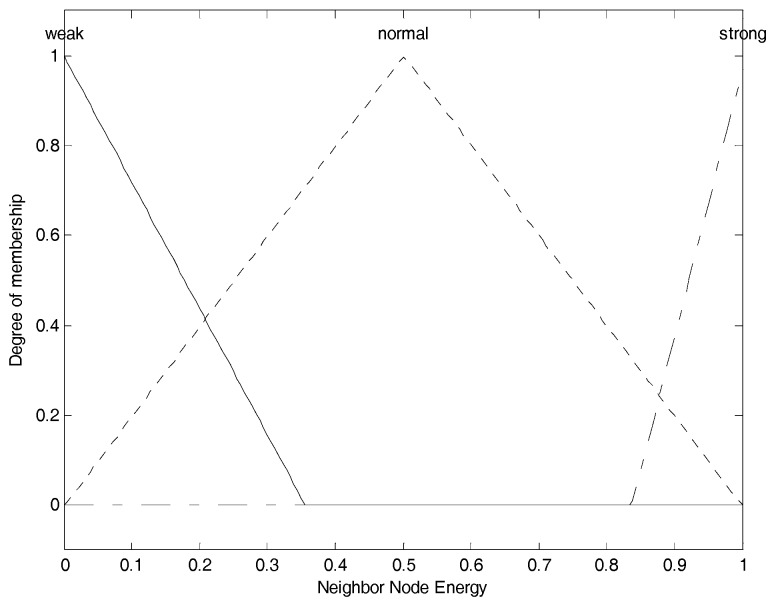
Neighbor nodes’ energy membership function.

The most widely used membership functions for fuzzy inference system are triangular membership function shown in Equation (8) and trapezoidal membership function shown in Equation (9). $\mu_{TRI}(x)$ and $\mu_{TRA}(x)$ stand for the membership degree in their respective membership function to describe the dynamic change of corresponding fuzzy linguistic variable. $a_{1}$, $b_{1}$ and $c_{1}$ are the mapping values on the *X* axis of the three vertices of the triangle respectively. $a_{2}$, $b_{2}$, $c_{2}$ and $d_{2}$ are the mapping values on the *X* axis of the four vertices of the trapezoid respectively. In general, the trapezoidal membership function is used for boundary variables and triangular membership function is used for intermediate variables [29]. Furthermore, by combining the membership function of each linguistic variable in the fuzzy set of the subordinate parameter, we can get the overall membership function for the corresponding parameter. These membership functions of input fuzzy parameters mentioned above are formulated by referring [30] as well as some of our own experimental experiences, which are shown in Figure 2, Figure 3 and Figure 4 respectively.

Those membership functions are used for the process of fuzzification. Figure 2 shows the membership function of node energy. The three membership functions for “*low*”, “*medium*” and “*high*” are, respectively, correspond to the fuzzy elements (“*low*”, “*medium*”, “*high*”) contained in node energy fuzzy set, and we use them to convert current crisp node energy into the corresponding membership degree set as the fuzzy variable. For example, assuming node energy is 0.3, corresponding to the “*low*” function, we can obtain the membership degree as 0.4; corresponding to the “*medium*” function, we can obtain the membership degree as 0.6; corresponding to the “*high*” function, also we can obtain the membership degree as 0. Thus, we can get the corresponding energy membership set as (0.4, 0.6, 0), which is the node energy fuzzy variable, and it can participate in the following further fuzzy logic analysis. This is also the same for Figure 3 and Figure 4. Figure 3 shows the membership function of node degree. The three membership functions for “*less*”, “*average*” and “*enormous*” are, respectively, correspond to the fuzzy elements (“*less*”, “*average*”, “*enormous*”) contained in node degree fuzzy set, and we use them to convert current crisp node degree into the corresponding membership degree set as the fuzzy variable. Figure 4 shows the membership function of neighbor node’s energy. The three membership functions for “*weak*”, “*normal*” and “*strong*” are, respectively, correspond to the fuzzy elements (“*weak*”, “*normal*” “*strong*”) contained in neighbor node energy fuzzy set, and we use them to convert current crisp neighbor node’s energy into the corresponding membership degree set as the fuzzy variable. The membership functions of “*low*”, “*medium*”, “*high*”, “*average*”, “*weak*”, “*normal*” and “*strong*” are the triangular and the membership functions of “*less*” and “*enormous*” are the trapezoidal. These specific shapes come from some of the existing conclusions and engineering experiences.

The segmented membership function for all the input fuzzy linguistic variables can be found in Figure 2, Figure 3 and Figure 4. The fuzzy inference system will convert the original crisp values to the fuzzy linguistic variables accordingly. Further, we can develop the IF-THEN rules in accordance to the mechanism of TSK deductive system.

The IF-THEN rules table of the EEDCF algorithm is shown in Table 2. NE stands for the residual energy, ND stands for the node degree, and NNE stands for the neighbor nodes’ average residual energy.

The fuzzy inference system (FIS) converts the original crisp input variables into corresponding fuzzy linguistic variables based on the input membership functions mentioned above. In the fuzzy logic field, the most widely used method to model human beings’ thinking is to make instantiation of linguistic expressions, such as the well-known IF premise, THEN conclusion, where the premise is the decision condition described by the fuzzy linguistic variables in the fuzzy set of input parameters, and the conclusion is regarded as the fuzzy output variable. The IF-THEN rule-based knowledge representation is based on natural language representations and models. It means that the fuzzy engine calculates the value of the output variables based on the rules which capture the experts’ knowledge of the evaluation systems [31]. The fuzzy rules and empirical data from our previous experiments are shown in Table 2. However, different from Mamdani method, the proposed TSK fuzzy system uses Equation (10) to calculate the crisp value as the consequence parameter instead of fuzzy linguistic variable.

(10) $$y^{i}(X)=p_{0}^{i}+p_{1}^{i}A_{1}(NE)+p_{2}^{i}A_{2}(ND)+p_{3}^{i}A_{3}(NNE)$$

The definition of TSK model output $\hat{y}$ is shown as Equation (11).

(11) $$\hat{y}=\frac{\sum_{j=1}^{m}\alpha_{j}y_{i}}{\sum_{j=1}^{m}\alpha_{j}}$$

where *m* is the number of fuzzy rules and $\alpha_{j}$ represents the membership function of the corresponding linguistic variable of fuzzy input parameter in the *j*th rule.

In order to facilitate the calculation, we define $\beta_{j}$ as Equation (12). Then, we get the following Equation (13) to describe the output $\hat{y}$.

(12) $$\beta_{j}=\partial_{j}/\sum_{j=1}^{m}\partial_{j}$$

(13) $$\begin{array}{ll} \hat{y} & =\sum_{j=1}^{m}\beta_{j}y^{i}(X)\\ & =\sum_{j=1}^{m}\beta_{j}(p_{0}^{i}+p_{1}^{i}A_{1}(NE)+p_{2}^{i}A_{2}(ND)+p_{3}^{i}A_{3}(NNE))\\ & =\;[\beta_{1},\;\beta_{1}x_{1},\;\cdots \;,\;\beta_{1}x_{n};\;\cdots \;;\;\beta_{m},\;\beta_{m}x_{1},\;\cdots \;,\;\beta_{m}x_{n}]\\ & \;\times {\left[p_{10},\;p_{11},\;\cdots \;,\;p_{1n};\;\cdots \;;\;p_{m0},\;p_{m1},\;\cdots \;,\;p_{mn}\right]}^{T} \end{array}$$

Combining the membership function of the three parameters (Node Energy (NE), Node Degree (ND) and Neighbor Node Energy (NNE)) and the previous experience examples in data base, we express the upper formula in a concise manner as shown in Equation (14).

(14) Y=XP

Then, we can get the least square estimation for *P* and further confirm the consequence parameter in TSK fuzzy model. Then we can calculate the probability of the final crisp output value $y_{TSK}(X)$ with Equation (15).

(15) $$y_{TSK}(X)\equiv \frac{\sum_{i=1}^{M}f^{i}(X)y^{i}(X)}{\sum_{i=1}^{M}\left(X\right)}$$

where $f^{i}(X)\equiv T_{K=1}^{P}\mu_{F_{k}^{i}}(x_{k})$, it means the rule’s excitation intensity, and *T* means *T*-norm.

After getting the crisp output, each node turns into compete CH state, sends Head_compete message including node’s ID and its own output to all its neighbor nodes within the communication radius “R”. Each node compares the output with its neighbors’ output. The node with less output turns into the member node state, and waits for a suitable cluster to join in after CH election; the node with higher output turns into the elected CH state as well.

### 4.3. Cluster Build Phase

The final elected CHs broadcast message CH_Message containing node ID within the communication radius “R”. The member nodes can estimate the distance to the CH node by received signal strength indication. A member node may receive more than one CH_Message from different CH nodes. If that happens, the node will choose the CH node with the shortest distance as the appropriate CH node and send Node_JOIN message to this CH node. Once receiving Node_JOIN message, the CH will return CH_ACCEPT message to the corresponding node to confirm its join request. Moreover, in the worst case, when there is no suitable CH within its communication radius “R” for a member node to join, it will elect itself as the CH node. Thus, we can finish the cluster formation process.

### 4.4. Message Complexity

At the beginning of every CH election, we assume the number of live nodes in current round of the network is *N*, and the number of Head_compete data package should be *N* too. After the comparison of the outputs between its neighbor nodes, we suppose *M* nodes are elected as CHs, they broadcast CH_Message to their neighbor nodes, and the number of these messages is *xM*. In the cluster build phase, the number of nodes which are not elected to be CH is (*N-M*), so accordingly the number of Node_JOIN message needed to be sent to the elected CH within the communication radius “R” is (*N-M*) as well. Meanwhile, the number of CH_ACCEPT messages which are responded from the corresponding CHs when the CHs receive the request of build cluster is also (*N-M*). The message complexity can be figured out with Equation (16), which calculates the total number of message transmissions ($N_{MT}$).

(16) $$N_{MT}=N+xM+2(N-M)$$

where *x* is the average node degree of all the CH nodes in current round. Thus, the overall message complexity of EEDCF is O(N).

## 5. Simulation and Analysis

We evaluated the performance of the proposed EEDCF algorithm by MATLAB simulation and compared with the EADEEG algorithm and DFLC algorithm in aspects of network lifetime, data transmission efficiency and system energy consumption respectively. To evaluate our scheme, two different scenarios were developed for the simulations. The area size of the two scenarios remains the same while the number of nodes in the network is chosen to be different. According to the network model and energy model in Section 3, we define the simulation parameters in the two scenarios in Table 3 [20,32]. As the nodes are randomly generated in the simulation, some coincidence factors might have some influence on the experiment results. Thus, we selected the average result of 50 experiments as the support to our conclusion in this paper.

**Figure 5:**
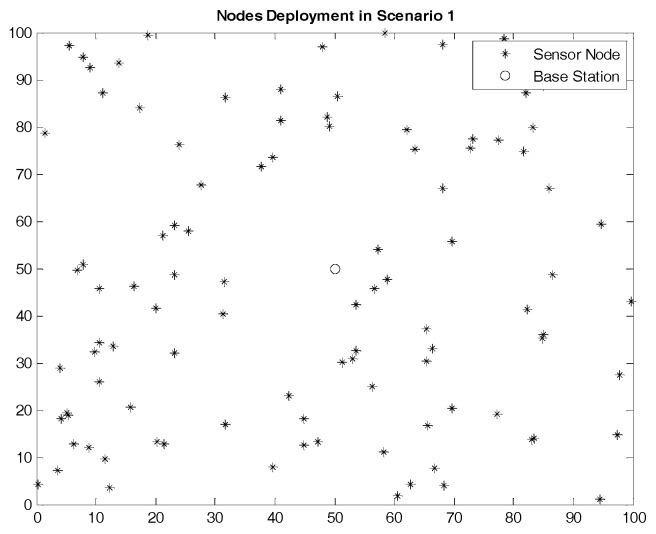
Nodes Deployment in Scenario 1.

**Figure 6:**
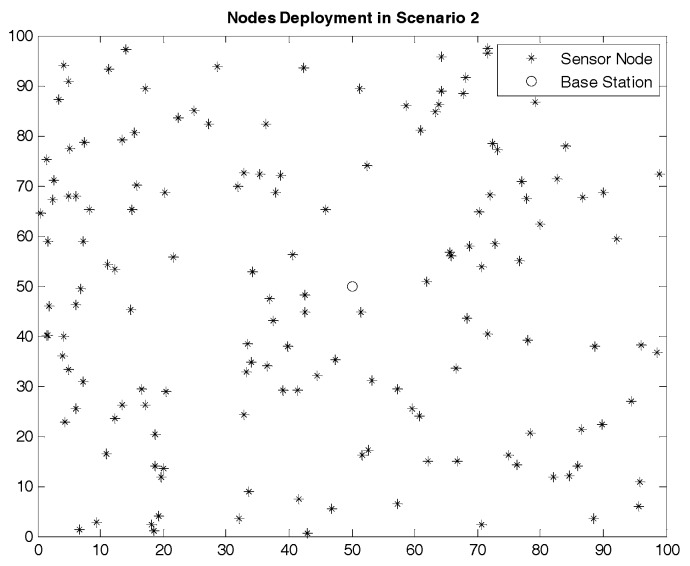
Nodes Deployment in Scenario 2.

The node deployment situations in the two scenarios are shown in Figure 5 and Figure 6. The region size of the two scenarios is the same while the node density in Scenario 2 is higher than that in Scenario 1. In other words, compared to Scenario 1, the number of nodes in each cluster in Scenario 2 is more intensive, and the communication condition is more complex.

**Figure 7:**
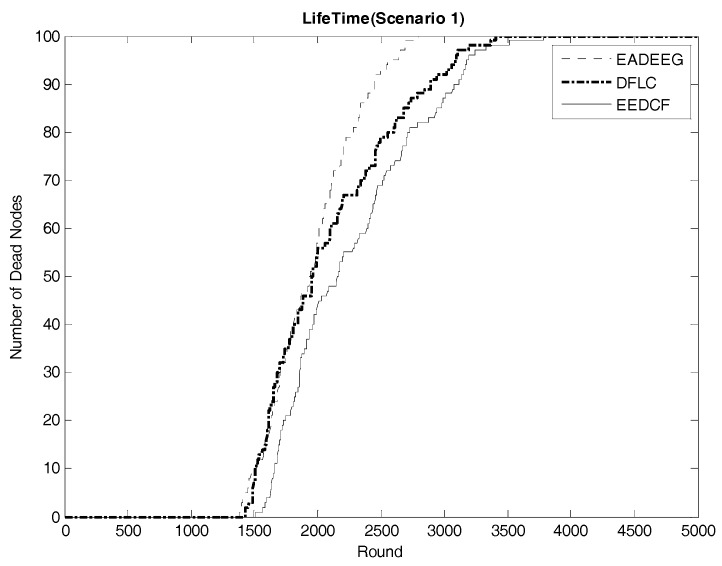
Comparison of network lifetime of the three algorithms in Scenario 1 (The vertical axis denotes the number of dead nodes, and the horizontal axis denotes the round number).

Network lifetime is a very important index to compare the performance of clustering routing protocol. There are various definitions to describe network lifetime of WSNs. The time at which half nodes die (HND), or all the nodes die (AND), in the network, can all be regarded as the network lifetime index. As the inflection point of network lifetime, the first node death (FND) destroys the integrity of the entire network, which also plays a significant role in network lifetime analysis. Figure 7 compares the network system lifetime of the three algorithms in Scenario 1. We can see that the FND of EADEEG algorithm is at 1382nd round, FND of DFLC is at 1429th round, and FND of EEDCF is at 1505th round. Because it is at the beginning of the death of nodes, the performance of the network system is not so bad, so the gaps among the three algorithms are not very obvious. As the rounds increase, the number of survival nodes becomes less and less, and the gap between the three algorithms will gradually enlarge. In Scenario 1, EADEEG algorithm runs out system energy (AND) in 2793rd round because it only considers the nodes residual energies and the distance from the nodes to BS in the phase of CH election. Different from EADEEG, DFLC and EEDCF take advantage of fuzzy logic system to make the measure of node’s performance more reasonable. DFLC takes node energy, node density, and distance to the base station as clustering factors which can avoid “isolate points” problem effectively, reduce extra energy consumption, and extend the network lifetime of AND to 3407 rounds. While the proposed EEDCF not only takes the nodes’ residual energies and degrees into account, but also introduces the neighbor nodes’ residual energies as the new input parameter additionally, it assigns the data forwarding tasks evenly to each node as much as possible, thus effectively alleviate the load balance problem in the network. The final network lifetime of AND by the proposed EEDCF is 3783 rounds, which is much better than the other two algorithms.

**Figure 8:**
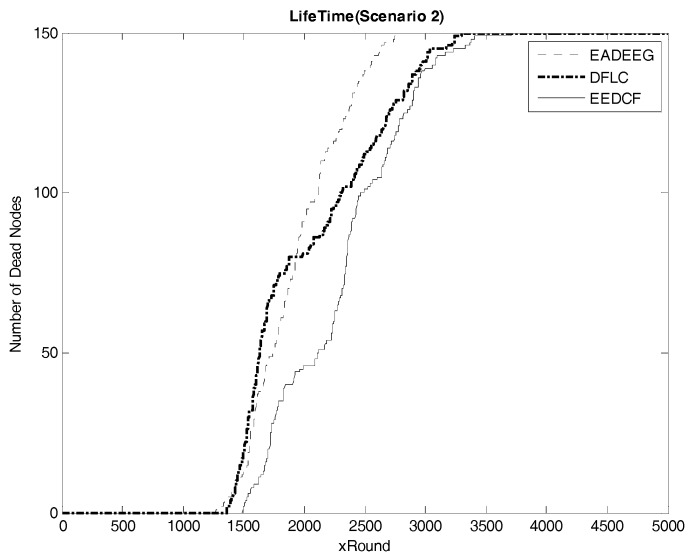
Comparison of network lifetime of the three algorithms in Scenario 2 (The vertical axis denotes the number of dead nodes, and the horizontal axis denotes the round number).

In Scenario 2, we increase the number of nodes in the same size region, which improves the node density of the whole network. Figure 8 compares the network system lifetime of the three algorithms in Scenario 2 and we can see that the FND of EADEEG algorithm is at 1270th round, FND of DFLC is at the 1359th round, while FND of EEDCF is at 1493rd round. The EADEEG again shows the worst performance due to the same shortages as we discussed in Scenario 1. As for the final round when all the nodes die, the AND of EADEEG is at the 2772nd round, the AND of DFLC is at 3299th round, and the AND of EEDCF is at 3712nd round. EEDCF algorithm still performs the best.

**Figure 9:**
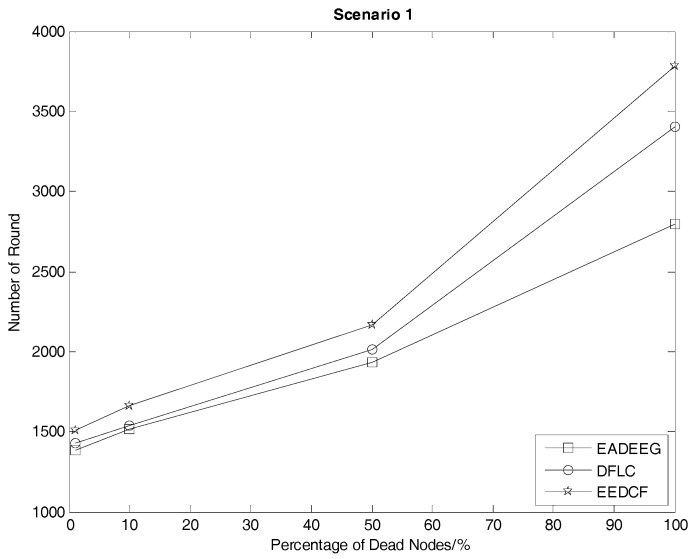
Number of round corresponding to different proportions of dead nodes of the network in Scenario 1.

**Figure 10:**
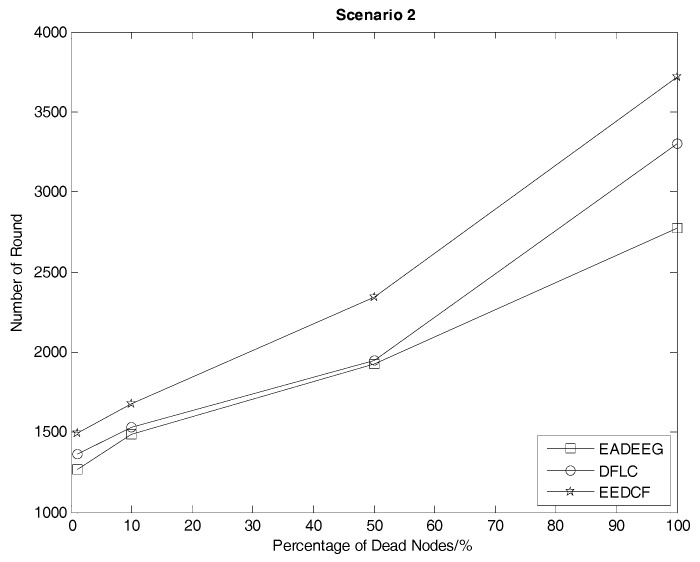
Number of round corresponding to different proportions of dead nodes of the network in Scenario 2.

According to the results in Figure 7 and Figure 8, we extract the specific number of rounds when some death occurs, such as the first node dies, half nodes dies and all nodes dies, in the two different scenarios, as shown in Table 4 and Table 5, to help us make a quantitative comparison and visualize the comparison, we plot those data into line graphs, as seen in Figure 9 and Figure 10, which show the performance gaps between the three algorithms more intuitively. As seen in Table 4, we consider HND metric as the evaluation index, and figure out the result that EEDCF is 11.6% more efficient than EADEEG and 7.7% more efficient than DFLC in Scenario 1. According to Table 5, we figure out that the performance of EEDCF is 21.8% better than EADEEG and 18.5% better than DFLC in Scenario 2. Comparing the experiment results in Scenario 1 and Scenario 2, we can conclude that the proposed EEDCF shows better performance than the other two algorithms, especially with more intensive network node deployment because of the additional consideration of the neighbor nodes’ residual energies.

Moreover, we can also see from the experimental results that, although considering the factor of distance between each node and BS in CH election can guarantee effective communication between CHs and base station, keeping the communication between each node and BS to get the distance will also increase the entire system energy consumption at the same time. In addition, in the variable and complicated topology in WSNs, it is also very hard for all the nodes to get the accurate distances with BS in real time. The proportion of CH nodes is not very large in the whole networks, and the elected CH node without considering the distance with BS may not be too far away from the BS, it is just a distribution with random probability. Thus, we can figure out that this energy consumption of CH election algorithm based on distances randomness will be smaller than those energy consumptions of the algorithm to keep all the nodes communication with BS in every round. In the distributed CH election situation, it does not require grasping all the precise information of the entire network. Hence, this kind of CH election based on distances randomness is an applicable method to be used in distributed networks. The proposed EEDCF does not choose the distance as the input of fuzzy variable, which may increase the possibility that some of the CHs are far away from the BS. Instead, this will economize the energy consumption for all the nodes communicating with BS in every CH election. From the experimental results, we can figure out that the consideration regarding the factor of distance from nodes to BS does not effectively reduce the overall energy consumption quite well in the implementation of the distributed clustering algorithm, especially for large scale networks.

**Figure 11:**
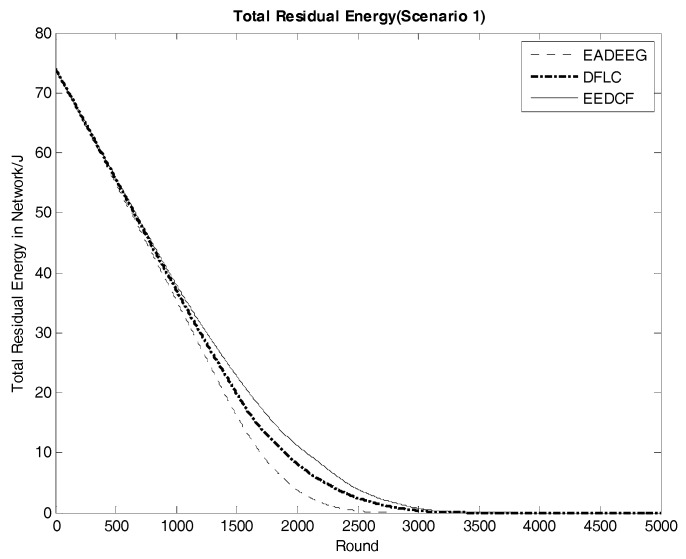
Comparison of system energy consumption of the three algorithms in Scenario 1 (The vertical axis denotes the energy of the system, and the horizontal axis denotes the round number).

**Figure 12:**
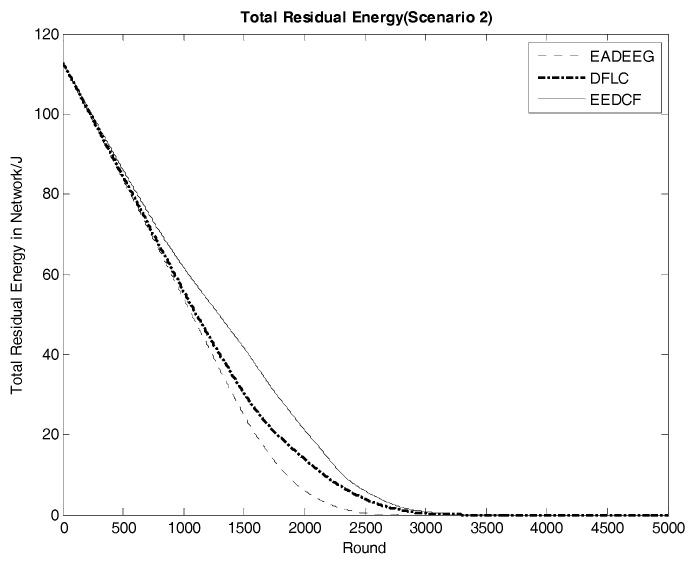
Comparison of system energy consumption of the three algorithms in Scenario 2 (The vertical axis denotes the energy of the system, and the horizontal axis denotes the round number).

Network energy consumption is a major factor in reflecting network performance. The system life cycle is also directly affected by the energy consumption of the networks. Figure 11 compares the energy consumption of the three algorithms in Scenario 1. From EADEEG to DFLC, and to EEDCF, the gradient of energy consumption curves is gradually slowing down, which is due to the usage of the fuzzy logic algorithm and increasingly reasonable optimization of CH election mechanism. We also extracted the real-time residual energy data of some designated rounds from Figure 11, and they are shown in Table 6. In the 1500th round, the total residual energy in EEDCF is 2.94 J more than that in DFLC, and 6.60 J more than that in EADEEG. In the 2000th round, the total residual energy in EEDCF is 3.05 J more than that in DFLC, and 7.44 J more than that in EADEEG. As we can see, the energy gaps between the three algorithms become bigger and bigger with the increase of round. Moreover, the proposed algorithm considers the load balancing problem in multi-hop communication mode, and introduces neighbor nodes’ residual energy as the additional fuzzy input variable, which can balance data forwarding pressure as much as possible to each sensor node in the network, and weaken the influence of the hotspots problem on the network performance. Compared to the two other algorithms, the proposed algorithm gets the lowest energy consumption finally. In Scenario 2, we can get almost the same analysis result from Figure 12, and the gaps of curves slope difference between the three algorithms in energy consumption are even more obvious when compared with that in Scenario 1. Then, we can figure out that, under the conditions of high node density, considering the residual energies of neighbor nodes can effectively optimize the network energy consumption. Further, Table 7 shows the real-time residual energy data of some designated rounds extracted from Figure 12 in detail.

**Figure 13:**
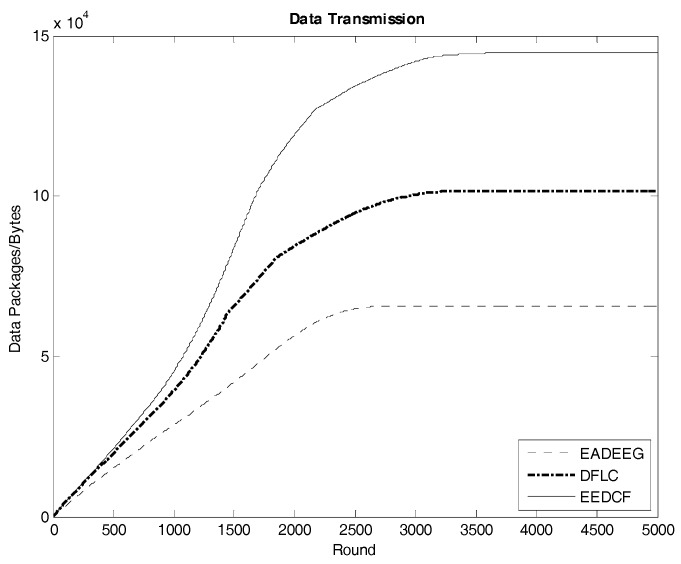
Data transmission in the network of the three algorithms in Scenario 1.

**Figure 14:**
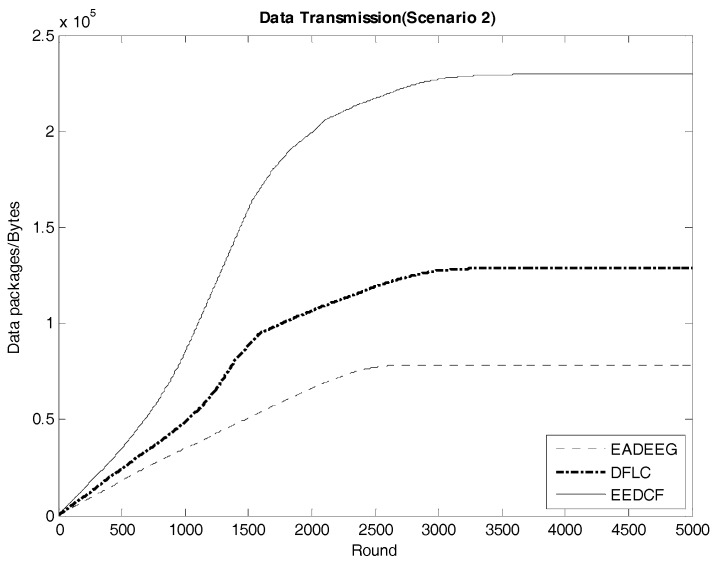
Data transmission in the network of the three algorithms in Scenario 2.

Figure 13 indicates the data transmission result of the three algorithms in Scenario 1. The EEDCF algorithm evenly divides the data fusion and data forwarding tasks in the cluster as much as possible to each node, which can improve load balance of the whole network and prolong the average lifetime of each node. Therefore, the EEDCF obtains the most effective data transmission among the three algorithms. Here we extract the specific total amount of package transmission within the setting rounds for the three algorithms in the two different scenarios, which is shown in Table 8. It can be seen that EEDCF and DFLC are better than EADEEG in data transmission efficiency due to both using of fuzzy logic mechanism and the additional consideration of the nodes density, which can effectively alleviate the unnecessary energy consumption caused by “isolate points” problem. Figure 14 indicates the data transmission result of the three algorithms in Scenario 2. We compare the final data transmission amount of the three algorithms in both scenarios in Table 8 and find that the relationship of the results in the two scenarios is very similar. In Scenario 1, the final result of data transmission in EEDCF is $14.7\times 10^{4}$ Bytes, which is 44.3% better than the result in DFLC, and 123.2% better than the result in EADEEG. In Scenario 2, the final result of data transmission in EEDCF is $22.9\times 10^{4}$ Bytes, which is 80.3% better than the result in DFLC, and 193.6% better than the result in EADEEG. Because the node’s density of Scenario 2 is higher than Scenario 1, it is reasonable, as shown in Table 8, that the data transmission amounts of the three algorithms in Scenario 2 are all higher than that in Scenario 1. Moreover, in the case of higher nodes density in the network, EEDCF algorithm improves the performance of the system even far better compared with DFLC algorithm and EADEEG algorithm, respectively. Certainly, the performance of DFLC is better than EADEEG because of the use of fuzzy inference system and the consideration of nodes density when CHs election occurring.

## 6. Conclusions

We propose a fully distributed clustering algorithm EEDCF, which better combines the clustering algorithm with the TSK fuzzy model. In the stage of CH election, we propose a scheme that not only considers the residual energy and node degree of the node, but also introduces the residual energy of the neighbor nodes as the new fuzzy input parameter. In addition, through the TSK fuzzy inference system, the probability that the node is currently elected as a CH node could be calculated reasonably. The proposed scheme improves the quality of CH candidate nodes to optimize CH election and solves the load balancing problem in multi-hop communication inside the cluster. The simulation results indicate that, compared with EADEEG algorithm and DFLC algorithm, the proposed EEDCF algorithm can prolong sensor nodes’ average life time, extend the life cycle of the whole network by reducing the energy consumption of the system, and improve the data transmission efficiency, which makes the whole system more energy efficient, especially for the networks with higher nodes densities. For the future works, we will further improve our algorithm aiming at security routing problems to try to establish the trust evaluation model by DS (Dempster–Shafer) evidence theory to defend the attacks from the malicious nodes in the network. In the stage of data communication within the cluster, we will strive to build the secure links between the nodes and CH, effectively reduce the end-to-end delay, and improve the performance of the whole network. All these efforts will make our research more suitable for practical applications.

## Figures and Tables

**Table 1:** Messages definition.

Message	Description
Node_MSG	Data package including node ID and residual energy
Info_table	Information table in each node
Head_compete	The node is in CH competing state
CH_Message	The node is selected as CH finally
Node_JOIN	Nodes failing to be elected as CH send this message to search for the closest CH to ask for joining
CH_ACCEPT	When new CHs receive Node_JOIN message from non-CH nodes, they return CH_ACCEPT message to finish the information docking

**Table 2:** IF-THEN rules.

List	NE	ND	NNE	Probability (%)
1	Low (0.31)	Less (3)	Weak (0.25)	0.26
2	Low (0.37)	Less (2)	Normal (0.47)	0.24
3	Low (0.28)	Less (4)	Strong (0.52)	0.36
4	Low (0.39)	Average (7)	Weak (0.21)	0.41
5	Low (0.41)	Average (9)	Normal (0.49)	0.46
6	Low (0.25)	Average (5)	Strong (0.78)	0.28
7	Low (0.15)	Enormous (10)	Weak (0.31)	0.16
8	Low (0.42)	Enormous (11)	Normal (0.50)	0.41
9	Low (0.33)	Enormous (13)	Strong (0.68)	0.24
10	Medium (0.56)	Less (4)	Weak (0.26)	0.42
11	Medium (0.48)	Less (4)	Normal (0.48)	0.46
12	Medium (0.63)	Less (5)	Strong (0.59)	0.51
13	Medium (0.67)	Average (8)	Weak (0.22)	0.58
14	Medium (0.59)	Average (6)	Normal (0.66)	0.69
15	Medium (0.71)	Average (9)	Strong (0.78)	0.76
16	Medium (0.66)	Enormous (9)	Weak (0.35)	0.61
17	Medium (0.53)	Enormous (9)	Normal (0.56)	0.62
18	Medium (0.44)	Enormous (11)	Strong (0.76)	0.51
19	High (0.66)	Less (6)	Weak (0.18)	0.59
20	High (0.73)	Less (3)	Normal (0.49)	0.47
21	High (0.76)	Less (2)	Strong (0.54)	0.43
22	High (0.81)	Average (4)	Weak (0.33)	0.68
23	High (0.58)	Average (7)	Normal (0.39)	0.69
24	High (0.89)	Average (9)	Strong (0.65)	0.83
25	High (0.72)	Enormous (8)	Weak (0.32)	0.76
26	High (0.61)	Enormous (11)	Normal (0.56)	0.78
27	High (0.76)	Enormous (9)	Strong (0.77)	0.81

**Table 3:** Simulation parameters.

Parameter	Scenario 1	Scenario 2
Sensor field	100 m×100 m	100 m×100 m
Number of nodes	100	150
BS location	(50, 50)	(50, 50)
Initial energy	0.5 ~ 1 J	0.5 ~ 1 J
$E_{elec}$	$5\times 10^{-8}\;J$	$5\times 10^{-8}\;J$
$\xi_{fs}$	$10^{-11}\;J$	$10^{-11}\;J$
$\xi_{mp}$	$1.3\times 10^{-13}\;J$	$1.3\times 10^{-13}\;J$
$E_{DA}$	$5\times 10^{-9}\;J$	$5\times 10^{-9}\;J$
Round	4000	4000
Data packet size	500 bytes	500 bytes

**Table 4:** Death time of nodes in some specific time points in Scenario 1.

	EADEEG	DFLC	EEDCF
First Node Dies/Round	1382	1429	1505
Teen Node Die/Round	1519	1537	1664
Half Node Die/Round	1943	2013	2168
All Node Die/Round	2793	3407	3783

**Table 5:** Death time of nodes in some specific time points in Scenario 2.

	EADEEG	DFLC	EEDCF
First Node Die/Round	1270	1359	1493
Teen Node Die/Round	1485	1534	1679
Half Node Die/Round	1922	1945	2341
All Node Die/Round	2772	3299	3712

**Table 6:** System residual energy in Scenario 1.

	EADEEG/J	DFLC/J	EEDCF/J
1000 Rounds	35.35	37.06	38.01
1500 Rounds	16.13	19.77	22.73
2000 Rounds	3.79	8.18	11.23
2500 Rounds	0.31	2.47	3.97

**Table 7:** System residual energy in Scenario 2.

	EADEEG/J	DFLC/J	EEDCF/J
1000 Rounds	54.61	55.76	61.71
1500 Rounds	24.79	30.22	41.59
2000 Rounds	6.10	13.97	21.17
2500 Rounds	0.42	4.01	6.03

**Table 8:** Data transmission in two scenarios.

	EADEEG/B	DFLC/B	EEDCF/B
Scenario 1	$6.58\times 10^{4}$	$10.2\times 10^{4}$	$14.7\times 10^{4}$
Scenario 2	$7.8\times 10^{4}$	$12.7\times 10^{4}$	$22.9\times 10^{4}$
